# Towards commercialization of fluorinated cation-disordered rock-salt Li-ion cathodes

**DOI:** 10.3389/fchem.2023.1098460

**Published:** 2023-01-13

**Authors:** Gi-Hyeok Lee, Jungwoo Lim, Jeongyim Shin, Laurence J. Hardwick, Wanli Yang

**Affiliations:** ^1^ Advanced Light Source, Lawrence Berkeley National Laboratory, Berkeley, CA, United States; ^2^ Department of Chemistry, Stephenson Institute for Renewable Energy, University of Liverpool, Liverpool, United Kingdom; ^3^ The Faraday Institution, Harwell Campus, Didcot, United Kingdom; ^4^ Umicore USA, Raleigh, NC, United States

**Keywords:** Li-ion batteries, cathode, cation-disordered rock-salt, commercialization, degradation pathway, surface coating

## Abstract

Cation-disordered rock-salt cathodes (DRX) are promising materials that could deliver high capacities (>250 mAh g^−1^) with Earth abundant elements and materials. However, their electrochemical performances, other than the capacity, should be improved to be competitive cathodes, and many strategies have been introduced to enhance DRXs. Fluorination has been shown to inhibit oxygen loss and increase power density. Nevertheless, fluorinated cation-disordered rock-salts still suffer from rapid material deterioration and low scalability which limit their practical applications. This mini-review highlights the key challenges for the commercialization of fluorinated cation-disordered rock-salts, discusses the underlying reasons behind material failure and proposes future development directions.

## 1 Introduction

Lithium-ion batteries (LIBs) are presently the go-to energy storage technology for electric vehicles and mobile devices. Performance enhancements over the past two decades, such as increases specific capacity and cycle life, have enabled wider use of applications. Goodenough and Park (2013); Yabuuchi (2018); Jin et al. (2020); Kobayashi et al. (2020); Lee et al. (2020b); Tian et al. (2021) However, the conventional layered intercalation materials are reaching their practical limits (∼250 mAh g^−1^). Thereby cathode structures with a high Li concentration are being actively sought. One such class of materials is the cation-disordered rock-salts (DRXs).

In LiTMO_2_ rock-salt type compounds, there are four types of crystal structures according to their cation ordering—DRXs, layered, spinel-like, and γ-LiFeO_2_. Other than the DRX structure, transition metal (TM) ions have preferred octahedral sites due to the crystal field stabilization energy, and it determines the ordering of the cations (Clément et al., 2018). As the TMO_6_ octahedra in rock-salts shares their edge, cation disorder inducing octahedral distortion is not energetically favored. On the other hand, an environment with low crystal field stabilization energy such as d^0^-centered octahedra, octahedral distortion is allowed so that cation disorder could happen (Urban et al., 2017).

Conventionally, the rock-salt-based crystal structure has not been considered as reversible ion hosts because of the absence of required Li-ion migration channels. However, an exception happens when the chemical composition is Li-excess, and the arrangement of cations is disordered (Lee et al., 2014). In this environment, a type of local structure is observed, which is named “0-TM” (Lee et al., 2014). Then, the percolation of Li-ion is enabled by the continuous connection of the 0-TM local structures. The discovery of the Li-ion percolation mechanism has led to significant attention to the DRXs (Yabuuchi et al., 2016; Cai et al., 2021; Ahn et al., 2022; Pei et al., 2022; Zhou et al., 2022). However, many thresholds must be overcome for the commercialization of DRXs, such as low cyclic retention, wide operation voltage, and unsuitable synthetic procedures.

Fluorination for DRXs requires the substitution of lattice oxygen by fluorine, and it is the most representative method to enhance the electrochemical performance of DRXs (Takeda et al., 2017; Campéon and Yabuuchi, 2021). As previously introduced by Ji et al., the intense amount of fluorine introduction significantly changes the physical properties of DRXs, and it leads to electrochemical behavior changes such as redox center rebalancing, redox potential modulation. Ji et al. (2019b), Clément et al. (2020) In general, when F is substituted for metal oxide materials, it is difficult to form a phase due to distortion of the coordination structure, and it limits a substitution concentration of F in oxides (Ménétrier et al., 2008). However, most of DRXs incorporate d^0^ transition metals (TM) to maintain a cation-disorder state as referred above. Urban et al. (2017) Thereby d^0^-centered octahedra eases the fluorination barrier so that relatively more significant amounts of fluorine can be substituted (Kitchaev et al., 2018). However, some aspects inherit or even worsen the negative aspects that DRX has for commercialization after fluorination. For example, synthetic difficulties for F substitution, side effects created additionally by F—F-loss, and contribution to the formation of Li-F short-range ordering (Ji et al., 2019a; Ji et al., 2019b; Crafton et al., 2020; Chen et al., 2021a).

Understanding the cause of failure mechanism enables determination of the stable operation limits of DRX whilst limiting material deterioration. For instance, the TM-dependent degradation process of F-DRXs suggests an essential criterion for designing the chemical composition of F-DRXs. Thus, this mini-review summarizes the thresholds for commercializing F-DRXs based on the latest reports and suggests a future research direction. The early part of the review summarizes the degradation process of F-DRXs, inherited from DRXs, and highlights the changes from the fluorination. The pros and cons of fluorination are discussed followed by an assessment of research directions to enable commercialization of F-DRXs.

## 2 Degradation of fluorinated cation-disordered rock-salts

**FIGURE 1:**
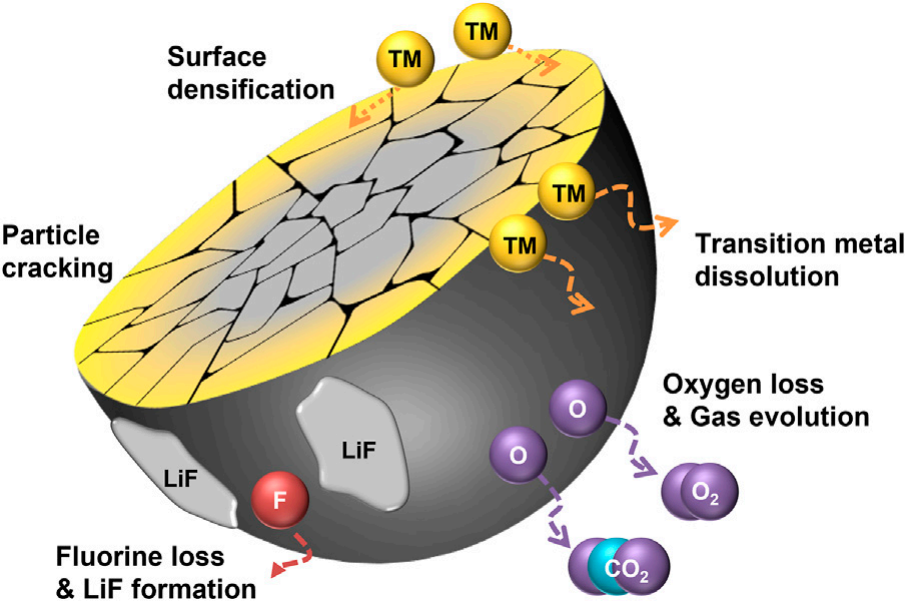
A schematic diagram illustrating the degradation pathways of fluorinated cation-disordered rock-salts (F-DRXs).

Most crystalline degradation of DRXs depend on their unstable surface character as shown in Figure 1, which illustrates the overall degradation process of F-DRXs. The deteriorated surface incorporates oxygen loss and TM migration (Lee et al., 2017; Crafton et al., 2020; Zhou et al., 2020; Huang et al., 2021; Schweidler et al., 2022). Although the causality between the oxygen loss and TM migration is not perfectly unveiled, the reduced TMs in the deteriorated surface phase suggest that the two phenomena are closely related. Therefore, it means that the instability of either the chemical stability of the lattice oxygen or the structural stability of the transition metal migration can contribute to the degradation.

First, the destabilization pathway through the oxidation of lattice oxygen in DRXs could be considered. Li-excess DRXs are known to have oxygen redox at the high state of charge, which means that the lattice oxygen is oxidized during the charging process (Seo et al., 2016; Zhou et al., 2020). The oxidized oxygen species in bulk could be stabilized by a specific coordination structure or chemical composition, and it retains partial reversibility by the reduction of the species (House et al., 2018; Eum et al., 2022). However, unlike bulk lattice oxygen, oxidized surface oxygen produces high reactivity by forming radicals due to the deficiency of coordination number. The oxidized surface oxygen reacts with the electrolyte to form CO_2_ or O_2_ gases, which are irreversibly lost (Lee et al., 2017; Crafton et al., 2020; Schweidler et al., 2022). Although the electrolyte decomposition at the cathode-electrolyte interface by the electron transport from highest occupied molecular orbital (HOMO) of electrolyte to cathode is reported in the Li-stoichiometric environment as well, the oxidized oxygen catalyzes the side reaction (Delmas et al., 1997). Also, recently reported oxygen redox in Li-stoichiometric layered oxide cathodes suggests the necessity to revisit the existing electrolyte decomposition mechanism from the perspective of the oxygen redox (Li et al., 2019; Lee et al., 2020a; Hu et al., 2021; Lee et al., 2021).

TM evolution with oxygen loss in DRXs is different from other layered oxide cathodes that are known to show oxygen redox. The degradation of the layered oxide surface proceeds from layered structure (TM:O = 1:2) to spinel structure (TM:O = 3:4) and finally transforms to rock-salt structure (TM:O = 1:1). Jung et al. (2014) The trend indicates the decrease in TM coordination number with oxygen, it has been interpreted that the transformation happens with the loss of oxygen (Boulineau et al., 2013; Shin et al., 2018; Cui et al., 2020). This phenomenon is called “densification” as the concentration of the surface increases. However, the crystalline structure of DRXs has rock-salt symmetry; most DRXs retain rock-salt structure after deterioration. It is noteworthy that the phase transition has transition metal dependence, Kwon et al. (2020).

The site previously occupied by Li^+^ gets emptied after the charging process, and surface transition metals migrate from the surface fill the site. Although the surface phase maintains the original symmetry, TM migration is a crucial structural degradation mechanism from the perspective of percolation, which is the fundamental Li-ion transport mechanism of DRXs. As also referred to in the introduction, Lee et al. suggested a “0-TM” local structure, an oxygen-centered tetrahedral coordinated structure surrounded by the Li-ion (Lee et al., 2014). The percolation happens through the continuous connection of the 0-TM. The Li-excess composition is necessary to percolate the Li-ion, however, filling the Li site with TM destroys the Li-excess environment at the surface. It blocks the percolation pathway and deters Li-ion transport.

In terms of preventing the loss of oxygen, fluorination has the advantage of minimizing oxygen oxidation by reducing the transition metal in DRXs (Lee et al., 2017; Crafton et al., 2020; Zhou et al., 2020; Huang et al., 2021; Schweidler et al., 2022). While the oxidation state of lattice oxygen is 2-, fluorine’s oxidation state is 1-, so fluorination allows TM to have more d electrons by reduction. In general, the charge compensation for the Li-ion extraction preferentially happens through d orbitals because the d orbitals of TMs are closer to the Fermi level than oxygen (Manthiram, 2020). As a result, TM reduction prevents the deterioration from oxygen oxidation. However, it is noteworthy that the reduced transition metals seem to not reach their original limits of the oxidation state after the charging process. In Ni-Ti-Mo-O-F DRXs compounds, soft X-ray absorption spectroscopy (sXAS) revealed that Ni^2+/4+^ redox reservoir is incompletely used (Lee et al., 2017). The Ni *L*_3_-edge spectra collected by the bulk-sensitive total fluorescence yield (TFY) mode showed that a significant amount of Ni^2+^ still exists at the fully charged state. Based on the nuclear magnetic resonance (NMR) spectroscopy and calculation results, Clement et al. claimed that Ni directly bonded with F is reduced to 2+, and F-neighboring Li is partially inaccessible due to the strong Li-F bonding, which is detail explained in the later part (Clément et al., 2018; Kitchaev et al., 2018). Nevertheless, the oxidation state of Ni evaluated by sXAS is much lower than other cathodes having Ni^4+^ and is still remained ambiguous. Hence, it requires a systematic sXAS study to elucidate the effect of fluorination on the TM redox centers, including Ni.

The stabilization of surface oxygen has been observed *via* diverse characterization. *Operando* online electrochemical mass spectroscopy (OEMS) shows a significant decrease in gas evolution like O_2_ or CO_2_ (Crafton et al., 2020). OEMS results of DRX with oxygen isotope substituted DRXs revealed that the oxygen contained in the gas came from the electrolyte instead of the material, suggesting the effect of fluorination prevented oxygen loss. In terms of spectroscopy, various techniques like resonant inelastic X-ray scattering (RIXS), XAS, X-ray photoelectron spectroscopy (XPS), electron paramagnetic resonance (EPR), and Raman spectroscopy have been used to detect oxidized oxygen species (Yamamoto et al., 2019; Chang et al., 2020; Mozhzhukhina et al., 2020; Naylor et al., 2020; Zhou et al., 2020). RIXS can detect a unique peak for the oxidized lattice oxygen; the intensity comparison of the unique peak can be used to check the reversibility of the oxygen redox (Yang and Devereaux, 2018). The fluorinated group showed higher peak intensity than the control group, and the reversibility at the extended cycles was also better than the control group. Besides, spectroscopies providing surface information like XPS and XAS with electron yield mode suggested that the surface TM reduction of the long-term cycling was enhanced after the proper amount of fluorination. The electrochemical performances correspond to the observations above, supporting that surface stabilization by fluorination prevents material degradation.

The reactive surface of DRXs also causes the TM dissolution issue, and it has been known that DRXs have relatively low surface stability while the surface of other cathode materials is passivated by CEI formation. Moghadam et al. had a report about the effect of fluorination to prevent TM dissolution in DRX with Li-Mn-Ti composition (Shirazi Moghadam et al., 2021; Shirazi Moghadam et al., 2022). Conversely, the V-containing surface layer of Li_2_VO_2_F was not passivated, and vanadium was steadily lost from the material by repeating the (de)formation during the cycling (Källquist et al., 2019; Källquist et al., 2020). Therefore it remains undetermined whether or not fluorination has an effect of TM dissolution in DRXs.

From the overall viewpoint, a significant degradation pathway of DRXs is particle cracking. As the cracking exposes the unreacted fresh surface to the electrolyte, it should be carefully understood to prevent surface degradation. Unlike the cracking of layered oxide cathodes, including Li-stoichiometric or Li/Mn-rich oxides, which gradually happens during the long-term cycling, immediate cracking happens in DRXs during their first cycle. Recently, Wang et al. suggested that DRX is a set of randomly oriented O3-packing blocks based on layered-like anisotropy (Wang et al., 2022). Although the study does not include any mechanical insights, the discovery of random anisotropy suggests that DRXs may weaker against the internal stress (Ortiz and Suresh, 1993; Lim et al., 2017). Interestingly, fluorination seems to affect the cracking behavior of the DRXs. Chen et al. reported that the cracking of DRXs is non-directional, fluorination leads to the aligned cracking by increasing the concentration of Li on (001) plane (Chen et al., 2021b). However, it is difficult to rule out the possibility that the improved electrochemical performance is due to other effects of fluorination. Even though the cracking pattern is significantly changed, the exposure of the new surface is resultantly the same. However, considering the reports from other cathodes, the reactivity between the newly formed surface and electrolyte might be different due to the facet preference, and reassembling behavior of pulverized particles could be different. Thus, further understanding of the relationship between directional cracking and its electrochemical performance is required.

## 3 Additional issues from fluorination

Fluorination is used as a method to minimize the failure of DRXs, and it provides electrochemical enhancements. However, the physicochemical property changes along with the substitution by fluorine leads to alternative side effects, which have to be understood and managed. Especially, the high thermodynamic stability of LiF lowers the solubility of F in DRXs, making LiF easily segregated during the fluorination, and it affects the overall degradation process of F-DRXs (Ménétrier et al., 2008; Szymanski et al., 2022).

F-DRXs show different densification behavior from DRXs. The densification of DRX includes the cationic arrangement with oxygen loss. Chen et al. reported that the additional anion sublattice rearrangement was named “anionic densification” for F-DRXs (Chen et al., 2021a). The F K-edge XAS with surface-detective total electron yield (TEY) mode was used for the characterization. The change of spectral shape indicated the change of F-containing species on the surface of F-DRXs, which means that the LiF-like domain is dominant at the surface as the cycle proceeds. Anionic densification in F-DRXs results in additional electrochemical performance degradation by forming an insulating LiF-like surface component.

Fluorine concentration increase at the surface by anionic densification means the total decrease of F concentration of the material. Besides the LiF-like domain formation, F-loss by dissolution has been reported (Crafton et al., 2020). OEMS helps quantify the dissolved fluorine by detecting gaseous trimethylsilyl fluoride. The addition of fluorine scavenger additives like tris(trimethylsilyl) phosphate (TMSPa) makes gaseous trimethylsilyl fluoride by reacting with dissolved F^−^. Crafton et al. reported the loss of F *via* dissolution at the high redox potential region based on the OEMS method.

Likewise, fluorine-loss through several pathways makes it difficult to retain the intended concentration of fluorine. The decrease in concentration may remove the benefits of fluorination. The continuous LiF formation, along with the cycle repetition, suggests that the degradation is not passivated at the early cycles. In addition, exposing a new surface from the particle pulverization implies that the degradation from fluorine could be accelerated.

The high energetic stability of LiF raises concerns not only for the surface, but also for the bulk. As demonstrated by Kitchaev et al., the strong Li-F binding limits the number of accessible Li to 0.4–0.8 per F (Kitchaev et al., 2018). In addition, a tetrahedrally coordinated Li-F short-range order is formed during the charging process. Clément et al. (2018) Li-F short-range order partially blocks the percolation pathway by forming a locally isolated Li cluster. On the other hand, interestingly, Ouyang et al. reported that Li-F short-range ordering is linked when the F content is increased above a certain amount, creating a new percolation pathway based on their theoretical calculation (Ouyang et al., 2020). Although it is experimentally validated, however, the demonstration was indirectly conducted by comparison of electrochemical profiles along with the F contents in the sample. Therefore, it is hard to confirm that continuous Li-F short-range order is critical for enhancing Li-ion transport. Therefore, the Li transport property per change of F contents requires a deeper understanding of theoretical and experimental studies.

## 4 Requirements for commercialization of fluorinated cation-disordered rock-salts

### 4.1 Artificial interphase formation at the material surface

Fluorination prevents the deterioration of materials by reducing the amount of oxygen loss, as summarized in part 2. However, it does not mean a complete removal of degradation factors, and introducing fluorine in DRXs also brings new degradation factors. Phenomenologically, the degradation that remains after fluorination of DRXs can be summarized as follows: 1) Blocking the Li percolation pathway by a concerted densification mechanism on the surface; 2) Decreased electric conductivity by LiF-like domain formation; 3) Increased CO_2_ evolution instead of decreased O_2_ evolution; 4) TM and fluorine loss.

**FIGURE 2:**
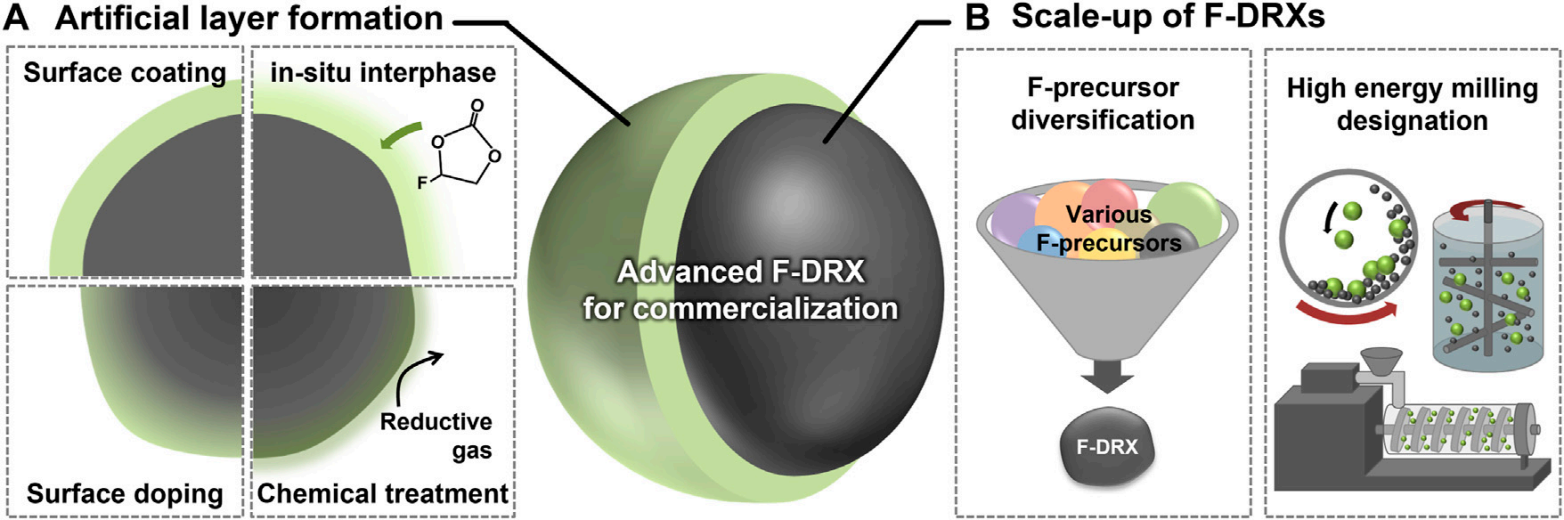
A schematic diagram suggesting the future research direction of fluorinated cation-disordered rock-salts (F-DRXs) to enable commercialization. **(A)** Methodologies for artificial interphase formation on the surface. **(B)** Requirements for the scale-up of F-DRXs including F-precursor diversification and designation of large-scale high-energy milling.

Unsurprisingly, most degradation except Li-F short-range ordering has arisen from their surface. Current commercial cathodes have introduced an interphase, which can prevent the material surface from direct contact with the electrolytes, to overcome the issues from unstable surfaces, and this has been shown to be a successful strategy. There are many ways to form the interphases, including *ex-situ* coating, *in-situ* interphase formation using electrolyte additives, surface doping, and chemical treatment as illustrated in Figure 2A (Choi et al., 2019; Huang et al., 2019; Lim et al., 2019; Naylor et al., 2020; Kim et al., 2021). The methods are not limited by their scalability and have an advantage from the perspective of commercialization. However, the existence of F at the surface of F-DRX differs their chemical properties from the other oxide materials. Thus, optimization of precursors and synthetic environments is required for the conformal interphase layer. In terms of process, the use of processes that can stimulate unstable F, such as heat treatment, is limited (Takeda et al., 2017). Thus, processes that can realize newly developed precursors within a lower synthetic temperature are needed.

### 4.2 Scale-up of fluorinated cation-disordered rock-salts

The synthetic procedure of F-DRX has been limited due to the low solubility of fluorine in oxides, and the mechanochemical synthesis is the only successful method so far. Although the mechanochemical synthesis is industry-friendly and scaleable, F-DRXs require high-energy ball milling like planetary ball-mill (Yabuuchi, 2022). Unfortunately, the scale of the planetary ball mill is structurally limited to hundreds of grams, which is a threshold for commercialization (Zhang et al., 2021). Therefore, the scalability enhancement of mechanochemical synthesis has the highest priority among the targets for commercialization until the emergence of the alternative synthetic procedure.

There are various issues to be addressed to overcome the synthetic issue of F-DRXs. First, a deeper understanding of the mechanochemical substitution of F through ball-milling (Chen et al., 2015; Cai et al., 2022). High-energy milling synthesis methods with higher production capacity include Simoloyer^®^, eccentric miller, and extruder, but the working principle, such as mixing and pressing method, is different depending on the design difference as shown in Figure 2B (Amrute et al., 2021; Colacino et al., 2021). Therefore, a correct understanding of the F-substitution enables the correct designation of the milling process. For example if, the instantaneous high pressure generated from the ball’s kinetic energy inside the planetary ball milling is the most important factor for F substitution. Then, the instrumentation used for scale-up must also have a similar pressure-generating mechanism. Next, the diversification of the F precursors is needed. Currently, LiF is the only successful precursor for F-DRXs (Szymanski et al., 2022). Although LiF is an excellent precursor, simultaneously providing Li and F without further impurity, the flexibility of the procedure can be limited. In addition, an additional variable, such as Li contents, happens if the targeting F contents change. If another phase of F precursor is developed, it will enhance the flexibility of the procedure. For example, discovering a gaseous F precursor could enable a gas-solid mechanochemical reaction. Of course, a careful experimental design should be followed, considering the environmental effects of fluorine precursor and safety.

## 5 Conclusion

The application of fluorination to DRXs has a high potential due to its high specific capacity. As summarized in this paper, fluorination seems beneficial to suppress the previously unstable surface reactions of DRXs, which may be a major origin of cycle life enhancement. However, there are still many hurdles to overcome in order to improve the material to a level for commercialization. For example, problems caused by the introduction of new elements such as LiF-like domain formation, difficult synthesis process, and surface side reactions that are not perfectly controlled. Therefore, additional research on new processes, the development of precursors, and methods applicable to industrial-scale processes such as surface coating are required. Ultimately, research on improving compatibility is needed when fluorination is applied with other strategies that improve the performance of DRXs itself.
